# Prevalence of human papillomavirus infection and genotyping for population-based cervical screening in developed regions in China

**DOI:** 10.18632/oncotarget.11498

**Published:** 2016-08-22

**Authors:** Yanru Zhang, Yueyun Wang, Li Liu, Chun Guo, Zhihua Liu, Shaofa Nie

**Affiliations:** ^1^ Department of Epidemiology and Biostatistics, School of Public Health, Tongji Medical College, Huazhong University of Science and Technology, 430030 Wuhan, Hubei, P.R. China; ^2^ Shenzhen Maternity and Child Health Hospitals, 518000 Shenzhen, Guangdong, P. R. China

**Keywords:** human papillomavirus, prevalence, genotype, cervical screening, China

## Abstract

We conducted a cross-sectional analysis to assess the distribution of human papillomavirus (HPV) types and explored an acceptable strategy for cervical screening in Shenzhen, China. A total of 2717 individuals ranging in age from 30–59 years were recruited. Clinical sensitivity and specificity as well as positive (PPV) and negative (NPV) predictive values were estimated. A triage strategy was regarded as acceptable when the NPV was at least 98.0%. 432 (15.9%) participants presented HPV positive. The five most prevalent HPV types were HPV52 (22.9%), HPV16 (12.7%), HPV53 (10.0%), HPV51 (8.6%), and HPV58 (8.1%). The CIN2+ risks for each HPV type were 40.0% for HPV33, 32.4% for HPV16, 18.2% for HPV58, 13.3% for HPV56, and 11.1% for HPV68 in descending order. Baseline cytology testing combined with HPV16/33/52/58 genotyping met the NPV thresholds at 98.6% with a PPV of 17.9%, demonstrating excellent clinical performance for detecting HPV types in CIN2+ patients. In conclusion, triaging HPV-positive women by baseline cytology combined with HPV16/58/33/52 genotyping is an acceptable strategy for cervical cancer screening in Shenzhen, China.

## INTRODUCTION

Human papillomavirus (HPV) infection is the primary risk factor for cervical cancer [1–3]. Several longitudinal studies have demonstrated that being positive for high-risk types of HPV is a predictor of cervical intraepithelial neoplasia [4]. And the results of several randomised clinical trials have demonstrated that the effectiveness of cervical cancer screening can be improved by detecting high-risk HPV DNA as a primary screening method for cervical cancer [5–10]. Because HPV subtypes vary in their carcinogenic potential, genotyping is necessary for the triage of HPV-positive women. In addition to the most common types, HPV16 and HPV18, other high-risk HPV types must be accurately detected. Therefore, the information regarding the HPV prevalence and type distribution in a given population is necessary for the cancer prevention with prophylactic HPV vaccines and the development and evaluation of HPV screening tests.

Several studies have shown that HPV testing is more sensitive than cytology for identifying women with high grade disease [9, 11–16], and these results support the use of HPV detection as a single primary screening test. Many countries, including the United States, are introducing HPV DNA detection for primary screening [17]. However, HPV testing detects more transient infection than cytology, which may result in over referral for colposcopy and ultimately overtreatment [14, 18, 19]. Therefore, management of HPV-positive women is of major concern, especially in regions with an inefficient cytology-based screening program [18].

Shenzhen, a newly-emerging economic developed city in China, is short of medical professionals for its short history and low experience. Since 2005, Shenzhen is among the first demonstrative bases of cervical cancer prevention and control in China. It offers a favourable environment for performing cervical screening, and is financially capable of covering the screening costs of the entire population. Thus, HPV testing is widely used as a primary screening method of cervical cancer in Shenzhen. However, information on prevalence and type distribution of HPV in Shenzhen is incomplete. In addition, Shenzhen has insufficient medical infrastructure for cytology-based screening and lack well trained cytology experts. Therefore, it is not clear which screening strategy is optimal in Shenzhen, co-testing or primary HPV detection followed by cytology for HPV-positive women. Furthermore, because HPV genotyping has been recommended for screening triage, it is uncertain which combinations of high-risk HPV types provide useful information for clinical practice in Shenzhen, China.

To address these concerns, we conducted a population-based cervical screening survey to assess the distribution of HPV genotypes in Shenzhen, China, and explore an acceptable triage strategy to reduce the burden of cytological examination.

## RESULTS

### Demographic and clinical characteristics of the studied population

Of the 3000 eligible women recruited to participate in the study, 283 exited for invalid questionnaire or unwilling participation of screening. We therefore based our analyses on the availability of complete data with HPV testing results for 2717 women (90.6%). The main characteristics of the study participants are shown in Table 1. The mean age was 40.6 years (standard deviation, SD 7.9; range 30–59), and the mean age of first sexual intercourse and primiparity was 22.9 years(SD 2.9; range 14–40) and 25.3 years (SD 4.1; range 14–46), respectively. The percentage of women using oral contraceptives was 2.7% (74 of 2717). The mean number of live births was 1.6 (SD 1.1; range 0–7). Married women accounted for 92.4% (2510 of 2717) of participants and 74.5% (2023 of 2717) had various forms of medical insurance (Table 1).

**Table 1:** Demographic information of the study population

Characteristics	n (%)	$\bar{\text{x}}\pm \text{s}$
Marital status		
Married	2510(92.4)	
Other	207(7.6)	
Age		40.6 ± 7.9
Age of first sexual intercourse		22.9 ± 2.9
≤ 16	13(0.5)	
17 ~ 20	581(22.8)	
≥ 21	1957(76.7)	
Age of primiparity		25.3 ± 4.1
≤ 20	154(6.2)	
21 ~ 25	1228(49.3)	
26 ~ 30	911(36.6)	
≥ 31	198(7.9)	
Number of live-births		1.6 ± 1.1
0	69(2.7)	
1	1287(50.6)	
2	857(33.7)	
≥ 3	328(12.9)	
Contraceptive measures		
Oral contraceptive	74(2.7)	
Intrauterine device	795(39.3)	
Tubal ligation	62(2.3)	
Condom	929(34.2)	
Rhythm method	86(3.2)	
Coitus interruptus	410(15.1)	
Other	361(13.3)	
Medical insurance		
Yes	2023 (74.5)	
No	694(25.5)	
HPV test		
Positive	432(15.9)	
Negative	2285(84.1)	
Liquid-based cytology		
NILM	1127(94.6)	
ASCUS+	64 (5.4)	
Histological diagnosis		
NILM	797(86.4)	
CIN1	95(10.3)	
CIN2+	30(3.3)	

HPV: Human papillomavirus; NILM: No intraepithelial lesion or malignant cells; ASCUS: Atypical squamous cells of undermined significance; CIN: Cervical intraepithelial neoplasia

**Figure 1:**
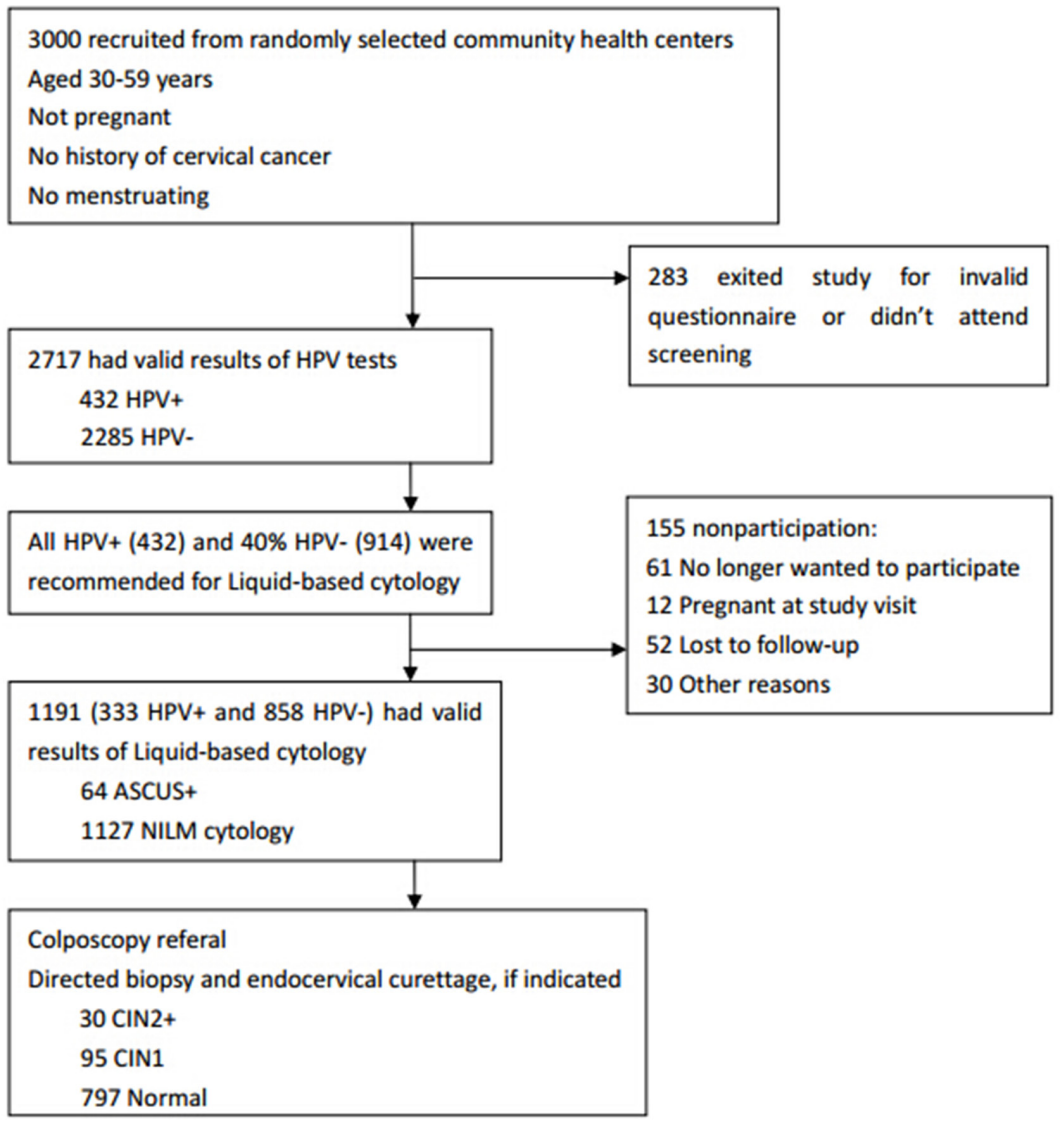
Flow diagram of procedures at every step of the study protocol

All study participants had valid HPV tests results, and 432 (15.9%) presented HPV positive. 333 HPV positive and 858 negative participants further underwent the cytology examination. The prevalence of abnormal cervical cytology (ASCUS+) was 5.4%. Among women who underwent liquid-based cytology testing, 922 were selected or randomly assigned for colposcopy. 30 women were diagnosed as CIN2+, and 95 were diagnosed as CIN1. (Table 1 and Figure 1)

### Prevalence and genotypes of HPV infections

**Figure 2:**
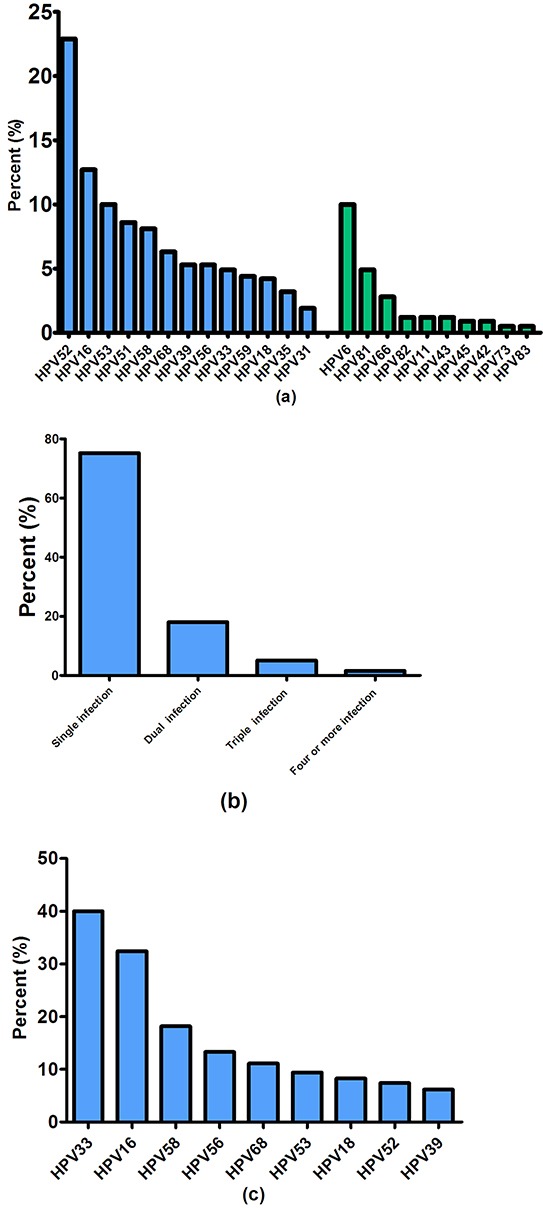
Prevalence and genotypes of HPV infections **Figure 2a.** Distribution of HPV genotypes in Shenzhen, China. The blue bar stands for the distribution of 13 high-risk HPV types, green bars stand for the distribution of other 10 HPV types; **Figure 2b.** Prevalence of single and multiple HPV infection; **Figure 2c.** Risk of CIN2+ for HPV genotypes in Shenzhen, China.

Positive HPV detection results were obtained for 15.9% (432) of the study participants. A total of 23 genotypes were detected among the HPV positive women, and the five most prevalent HPV types were HPV 52 (99/432=22.9% of HPV infections), HPV16 (55/432=12.7% of HPV infections), HPV53 (43/432=10.0% of HPV infections), HPV51 (37/432=8.6% of HPV infections), and HPV58 (35/432=8.1% of HPV infections). Women demonstrating positivity for a single HPV genotype accounted for 75.2% (325/432), whereas 24.8% (107/432) of women were positive for multiple types. Among the latter, 18.1% (78/432) had dual infections, 5.1% (22/432) had triple infections, and 1.6% (7/432) had four or more infections (Figure 2a and Figure 2b).

We also estimated the CIN2+ risks (positive predictive values and negative predictive values) for each individual HPV type. In Figure 2c, we showed these risks in descending order, from greatest to least. For HPV infected women, the risks of CIN2+ were 40.0% (95% CI: 15.2 - 64.8) for HPV33, 32.4% (95% CI: 18.0 - 49.8) for HPV16, 18.2% (95%CI: 5.2 - 40.3) for HPV58, 13.3% (95% CI: 1.7 - 40.5) for HPV56, and 11.1% (95% CI: 1.4 - 34.7) for HPV68.

### Distribution of HPV genotypes by sociodemographic, cytological, and histological diagnosis

Table 2 explored the relationship between sociodemographic, cytological, and histologic diagnosis and the distribution of HPV genotypes. We observed that the prevalence of HPV positivity was significantly lower in married women (385/2510,15.3%) than in single/divorced women(47/207,22.7%)(*P=0.005*). For the age of first sexual intercourse, the prevalence of HPV infection was significantly different among ≤16 years, 17~20 years, and ≥21 years (*P*=0.047), with the highest prevalence in women aged 17~20 years(18.2%) then followed by women aged≥21 years (14.3%) and ≤16 years(7.7%). HPV infection prevalence was significantly lower among women who have medical insurance (14.9%) than among those without (18.7%) (*P*=0.018). Table 2 also shows the prevalence of HPV genotypes according to cervical cytology and histological diagnosis. The HPV positive prevalence was 25.3% in NILM cases, whereas the prevalence was 75.0% in women with abnormal cervical cytology (*P*<0.001). HPV 16/58 prevalence was 2.9%/1.8% in NILM cases, whereas the prevalence was 17.2%/10.9% among cases of abnormal cervical cytology (*P*<0.001). Infection with single and multiple HPV types was noted in 62.8%/37.2% of NILM cases and 43.8%/56.2% of abnormal cervical cytology cases (*P*=0.013). As expected, HPV positivity increased with the severity of the pathological result(*P*<0.001). Moreover, the prevalence of HPV16/58 also exerted a similar increasing trend with the development of pathology abnormalities(*P*<0.001). The prevalence of single and multiple HPV infections was statistically different among CIN2+, CIN1, and NILM groups (*P*=0.029).

**Table 2:** Distribution of HPV genotypes by sociodemographic, cytological and histologic diagnosis

Characteristic	HPV (n, %)		HPV16 (n, %)	HPV58 (n, %)	Infection with single HPV type (n, %)	Co-infection with multiple types (n, %)
	HPV positive	HPV negative				
Marital						
Married	385(15.3)	2125(84.7)	48(1.9)	32(1.3)	246(63.9)	139(36.1)
Single/divorced	47(22.7)	160(77.3)	7(3.4)	3(1.4)	24(51.1)	23(48.9)
χ^2^	7.761		2.082	0.046	2.943	
*P* value	0.005		0.149	0.831	0.086	
Age of first sexual intercourse						
≤16	1 (7.7)	12(92.3)	0(0.0)	0(0.0)	1(100)	0(0.0)
17 ~ 20	106(18.2)	475(81.8)	15(2.6)	7(1.2)	64(60.4)	42(39.6)
≥21	279(14.3)	1678(85.7)	34(1.7)	24(1.2)	180(64.5)	99(35.5)
χ^2^	6.111		1.951	0.162	1.145	
*P* value	0.047		0.377	0.922	0.564	
Age of primiparity						
≤20	20(13.0)	134(87.0)	3(1.9)	2(1.3)	15(75.0)	5(25.0)
21~25	193(15.7)	1035(84.3)	22(1.8)	10(0.8)	121(62.7)	72(37.3)
26~30	122(13.4)	789(86.6)	13(1.4)	13(1.4)	77(63.1)	45(36.9)
≥31	27(13.6)	171(86.4)	3(1.5)	4(2.0)	20(74.1)	7(25.9)
χ^2^	2.765		0.538	3.138	2.414	
*P* value	0.429		0.910	0.371	0.491	
Parity						
Nullipara	11(15.9)	58(84.1)	1(1.4)	1(1.4)	5(45.5)	6(54.4)
Multipara	367(14.8)	2105(85.2)	45(1.8)	29(1.2)	236(64.3)	131(35.7)
χ^2^	0.064		0.052	0.044	1.642	
*P* value	0.801		0.820	0.834	0.200	
Contraceptive measures						
Oral contraceptive	14(18.9)	60(81.1)	2(2.7)	1(1.4)	10(71.4)	4(28.6)
Other contraceptive measures	418(15.8)	2225(84.2)	53(2.0)	34(1.3)	260(62.2)	158(37.8)
χ^2^	0.519		0.177	0.002	0.492	
*P* value	0.471		0.674	0.961	0.483	
Medical insurance						
Yes	302(14.9)	1721(85.1)	38(1.9)	27(1.3)	189(62.6)	113(37.4)
No	130(18.7)	564(81.3)	17(2.4)	8(1.2)	81(62.3)	49(37.7)
χ^2^	5.591		0.850	0.134	0.003	
*P* value	0.018		0.357	0.714	0.957	
Cytological						
NILM	285(25.3)	842(74.7)	33(2.9)	20(1.8)	179(62.8)	106(37.2)
ASCUS+	48(75.0)	16(25.0)	11(17.2)	7(10.9)	21(43.8)	27(56.2)
χ^2^	74.302		34.610	22.949	6.220	
*P* value	<0.001		<0.001	<0.001	0.013	
Histological diagnosis						
NILM	184(23.1)	613(76.9)	12(1.5)	9(1.1)	105(57.1)	79(42.9)
CIN1	83((87.4)	12(12.6)	14(14.7)	9(9.5)	59(71.1)	24(28.9)
CIN2+	28(93.3)	2(6.7)	11(36.7)	4(13.3)	13(46.4)	15(53.6)
χ^2^	214.819/194.228*		124.413/121.720*	41.330/40.160*	7.058	
*P* value	<0.001/<0.001*		<0.001/<0.001*	<0.001/<0.001*	0.029	

HPV 16 and HPV 58 were both among the five most prevalent HPV types (HPV52, 16, 53, 51, and 58) and the five most risk HPV types (HPV33, 16, 58, 56, and 68) in Shenzhen. Therefore, we explored the relationship between the distribution of HPV16 and HPV58 and sociodemographic, cytological, and histologic diagnosis in Table 2.

HPV: Human papillomavirus; NILM: No intraepithelial lesion or malignant cells; ASCUS: Atypical squamous cells of undermined significance; CIN: Cervical intraepithelial neoplasia

* Linear-by-Linear test

### Evaluation of triage strategies for high-risk HPV positive women

The performance of the eleven triage strategies for HPV positive women at baseline is shown in Table 3. The inclusion of HPV16 and HPV16/58, yielded an NPV for CIN2+ cases of 93.8% and 94.5%, respectively, and a PPV of 32.4% and 25.9%, respectively. Triage of HPV-positive women based on cytology testing yielded an NPV for CIN2+ of 95.9% and a high PPV of 39.1%. The inclusion of HPV16/33, HPV16/58/33, and HPV16/33/52/58 genotyping at baseline provided a slightly higher NPV (95.5%, 95.6%, and 96.4%) and a decreased PPV (33.3%, 25.7%, and 17.1%). The NPV of the above triage strategies were well below our threshold of 98.0%, and therefore these strategies were deemed unacceptable. The triage strategy of baseline cytology testing combined with HPV16, HPV16/58, HPV16/33, or HPV16/58/33 genotyping demonstrated an NPV of 97.0%-97.6%, which was slightly below threshold, and a PPV of 24.5%-31.0%. Only the baseline cytology testing combined with HPV16/33/52/58 genotyping met the NPV thresholds, with an NPV of 98.6% and a PPV of 17.9%. The sensitivity and specificity for detection of CIN2+ were 92.9% and 54.2%, respectively.

**Table 3:** Different triage strategies for high-risk HPV positive women

Triage strategy	HPV positive women			
	Endpoint CIN2+			
	NPV (95%CI),%	PPV (95%CI),%	Sensitivity (95%CI),%	Specificity (95%CI),%
Cytology	95.9(92.5,98.0)	39.1(25.0,53.2)	64.3(46.5,82.0)	89.2(84.8,92.7)
HPV16	93.8(90.1,96.4)	32.4(18.0,49.8)	42.9(24.5,62.8)	90.6(86.5,93.9)
HPV16/58	94.5(90.8,97.1)	25.9(15.3,39.0)	53.6(33.9,72.5)	83.9(78.9,88.1)
HPV16/33	95.5(92.1,97.7)	33.3(20.8,46.3)	60.7(42.6,78.8)	87.3(82.7,91.0)
HPV16/58/33	95.6(92.0,97.9)	25.7(16.0,37.6)	64.3(46.5,82.0)	80.5(75.3,85.1)
HPV16/58/33/52	96.4(92.3,98.7)	17.1(11.0,24.7)	78.6(59.1,91.7)	59.9(53.8,65.9)
HPV16 & Cytology	97.2(94.1,99.0)	31.0(20.5,43.1)	78.6(59.1,91.7)	81.2(75.9,85.8)
HPV16/58 & Cytology	97.0(93.7,98.9)	25.9(17.0,36.6)	78.6(59.1,91.7)	75.8(70.1,80.9)
HPV16/33 & Cytology	97.6(94.5,99.2)	28.8(19.2,40.0)	82.1(63.1,93.9)	78.1(72.6,83.0)
HPV16/58/33 & Cytology	97.4(94.1,99.2)	24.5(16.2,34.4)	82.1(63.1,93.9)	72.7(66.9,78.0)
HPV16/58/33/52 & Cytology	98.6(95.1,99.8)	17.9(12.1,25.2)	92.9(76.5,99.1)	54.2(48.2,60.3)

HPV: Human papillomavirus; CIN: Cervical intraepithelial neoplasia; NPV: Negative predictive value; PPV: Positive predictive value.

## DISCUSSION

This independent study evaluated the prevalence of HPV genotypes in Shenzhen, a developed coastal city in China. A total of 15.9% of the study participants presented HPV positive, which was lower when compared to some western countries such as America, Italy, and Canada (18.1%-39.0%) [20–23], and higher than that in India and other regions of China (6.1%-12.9%) [20, 24]. This indicates the cultural, ethnic and regional differences in HPV prevalence [25]. And various detection methods may also contributed to the difference [26].

Our present study showed that the prevalence of HPV positivity was significantly lower in married women (15.3%) than in single/divorced women (22.7%), which was in agreement with a study conducted in Italy [27]. The low prevalence of HPV infection among married women may be due to the protective effect against HPV infection generated by living with a partner [27]. For the age of first sexual intercourse, the prevalence of HPV positivity was higher among the group aged 17~20 years(18.2%) than among that aged≥21 years (14.3%) and ≤16 years(7.7%). In some regions, an age at first sexual encounter of younger than 17 years is generally associated with increased risk of HPV infection, and the same conclusion was draw in our study. Early age of sexual debut was a risk factor for HPV infection. It is reported that the immature cervix during adolescence is more likely to acquire HPV infection and therefore have a greater risk of precancerous lesions. Recent international studies describe a higher risk of acquiring new HPV infections at younger ages. These findings suggest that younger women and the associated risk of early sexual debut should be identified as potential targets for the prevention of HPV infection [28–31]. HPV infection prevalence was significantly lower among women with medical insurance (14.9%) than among those without (18.7%). This can be attributed to the fact that having medical insurance is associated with individual economic conditions, education level, and employment situation in China, and these factors are important determinants for HPV infection [27]. It is reported that oral contraceptives use was significantly associated with HPV infection, while some other studies have failed to confirm such an association [32, 33]. In our study, we didn't found the significant association between the use of oral contraceptives and HPV positivity. Considering that HPV infections are closely linked with the sexual habit, and the adopted sexual behaviours are in turn closely related to contraception use of individual women, it is of great necessity to consider the effect of HPV infection, sexual habits, and other confounding factors(such as barrier contraception and socio-economic factors) in the future research so as to promote the research process in this field [33]. The HPV positive prevalence was 25.3% in NILM cases, and 75.0% among ASCUS+ women. Moreover, HPV positivity increased with the severity of the pathological result (*P*<0.001). Sellors et al. reported similar results, demonstrating that the presence of squamous intraepithelial lesions was strongly associated with HPV infection [22]. In addition, Ozturk and colleagues reported that HPV infection together with high rates of precancerous lesions leading to malignancy supported the association between HPV and cervical cancer [20]. In our current study, the high prevalence of HPV infection in precancerous lesions indicates the need for cervical screening and follow-up in women who are HPV positive.

Our results showed that HPV 52, HPV16, HPV53, HPV51, and HPV58 were the five most prevalent types in this population, which is similar to studies of HPV prevalence in other countries in Asia [34], and in other regions in China [24, 35]. A meta-analysis of HPV prevalence in 5 continents displayed that HPV16 and HPV18 were the most frequent types worldwide, with HPV16 being the most common type everywhere, and HPV18 was among the most common HPV types after HPV16 [34, 36]. However, HPV 52 and HPV58 are more common in the Asian population [37, 38]. In China, HPV 52 and HPV 16 are more prevalent, although variation also exists among different regions: HPV52, 16, 58, 31 were the most common types in Shanghai, HPV52, 16, 58, 18, 56 in Yunnan province, HPV16, 58, 52, 33, 11 in Zhejiang province and HPV16, 52, 58, 18, 45 in Guangdong province [24, 39–41]. These differences in HPV type prevalence may be linked to geographic location and complex immune/genetics factors which would influence the biological interactions between host immune system and different HPV subtypes [42, 43]. In addition, HPV genotype distribution may also vary by ethnicity/race, which is related to socioeconomic status [44, 45].

We also examined the risks of CIN2+ for each individual HPV type. Long-term observational studies show that HPV16 and HPV18 have an elevated risk of cervical lesions compared to other high-risk HPV types [46–49]. However, genotyping triage for HPV16 and HPV18 is not suitable for China because of the varying HPV genotype distribution between China and other countries. Thus, there is a need to distinguish additional HPV types to guide cervical screening. For the HPV infected women in our study, the CIN2+ risks for each HPV type were 40.0% for HPV33, 32.4% for HPV16, 18.2% for HPV58, 13.3% for HPV56, and 11.1% for HPV68 in descending order. The prevalence of HPV genotypes in our study indicated that compared to bivalent (HPV16, 18) and quadrivalent (HPV6, 11, 16, 18) HPV vaccines, the 9vHPV (HPV6, 11, 16, 18, 31, 33, 45, 52, 58) vaccine is more suitable for the population of Shenzhen, China. Our results also allude to the direction of future vaccines most suitable for the Chinese population. We also observed that the proportion of HPV16/58 in ASCUS+ cases was significantly higher than that in NILM cases, and the prevalence of HPV16/58 exerted an increasing trend with the development of pathology abnormalities. Similar findings showed that infection with HPV16/58/31/33 may increase the risk for progression of cervical lesions in the study population [50]. Another study also reported a strong association between abnormal cytology and HPV16/18 infection [51].

The proportion of women with multiple infections among HPV-positive participants was 24.8%, and our results correlate with the findings of similar studies conducted in China, in cities such as Beijing (27.7%), Shanxi (24.3%), and Henan (19.8%) [52]. Studies show that the prevalence of multiple HPV infection ranges from 9%-50%[51], and the ratio mainly depended on the individual immune status of the study population and the method used for HPV genotype detection [53]. In addition, the prevalence of single HPV infection was significantly higher in married women than in single/divorced women, and this ratio is consistent with the proportion of overall single infections in our study. In the current study, the prevalence of multiple HPV infections was more common in ASCUS+ (56.2%) and CIN2+(53.6%) cases than in NILM cases (37.2% and 42.9%). In addition, the prevalence of single HPV infection was more prevalent in NILM (57.1%) and CIN1(71.1%) cases compared to CIN2+(46.4%) cases. Several studies have shown that multiple HPV infections increase the severity of cervical lesions, and may influence the oncogenic potential of HPV [54–59]. Therefore, the survey of multiple infections in our study enhances our understanding of the role of multiple HPV infections in cervical cancer development. Among the women infected with multiple HPV types, double and triple HPV infection were more common, and our results are in agreement with the findings of similar studies [53].

Implementation of HPV detection has been recommended as a primary screening test for cervical screening. However, the specificity of HPV testing is far below that of cytology examination, and results in over-diagnosis and over-treatment of HR HPV-positive women [9]. Therefore, triage strategies for HPV positive women are necessary. Based on the prevalence of HPV types in Shenzhen, we estimated different combinations of HPV types in the study to find an acceptable triage strategy for HPV positive women. We also evaluated the performance of eleven triage strategies for HPV positive women. The acceptable triage strategy was baseline cytology testing combined with HPV16/33/52/58 genotyping, which met the thresholds for NPV of 98.6% and had an acceptable PPV of 17.9%[18]. This can best balance the safety (NPV) and the burden on patients for an acceptable strategy [60]. In our study, readouts of all HPV types for the reverse dot blot HPV test are provided concurrently, and testing for HPV16/33/52/58 in the study to triage HPV positive patients was very efficient and greatly reduced the manpower requirements in the clinical. The triage strategy of our study also indicated that cytology could be applied to HPV16/33/52/58 positive women, which could relieve the burden of cytological examination. In addition, this strategy will increase the sensitivity for identification of CIN2+ in HPV positive women, while maintaining acceptable PPV. Similar studies in the United States showed that HPV genotyping (HPV16/18) with or without cytology can provide safe and cost-effective cervical screening and the trade-offs in sensitivity for detection of CIN3+ versus the poor PPV [15]. Another study found that baseline and repeat cytology testing for HPV positive patients demonstrates the highest PPV(34%) and a low referral rate. However, this triage does not apply to cities with less efficient cytology screening, such as Shenzhen [60].

Our study has the advantage of examining a wide age range of participants (30-59 years), which is encouraged for HPV testing. Furthermore, our study was conducted in a population subset, in which the results can be extrapolated to the larger Chinese population. This study also has limitations. For instance, as a baseline strategy, an important disadvantage is the limited PPV, which although in an acceptable range, suggests a considerable risk for overtreatment [60]. Further studies will need to evaluate the performance of prospective studies to identify best practices.

In summary, HPV52, HPV16, HPV53, HPV51, and HPV58 were the most prevalent HPV types, and HPV33, HPV16, HPV58, HPV56 and HPV68 were the most risk HPV types in Shenzhen. The ability to identify HPV16, HPV58, and the other 21 HPV types make the reverse dot blot HPV test a very attractive option for HPV detection, and potentially contribute to improve patient management. In the management of HPV-positive women, triaging HPV-positive patients by baseline cytology testing combined with HPV16/33/52/58 genotyping seems safe and yields an acceptable PPV. The weights placed on the quality of cytology and the safety of the screening ultimately determines the management effectiveness of HPV-positive women.

## MATERIALS AND METHODS

### Study population and procedures

For this study, we enrolled women who were able to give independent informed consent, aged 30 to 59 years, living in Shenzhen, Guangdong Province, China for more than six months, and screened them for cervical cancer from 1 March to 15 June 2015. Eight community health centres in Luohu district and ten community health centres in Nanshan district were selected using the randomised cluster sampling method. All eligible women from the eighteen selected community health centres were invited to participate. Non-pregnant women with no history of cervical cancer, precancerous lesions, hysterectomy, pelvic radiation, and other screening contraindications were eligible for enrolment. All participants provided written informed consent. The study was approved by the Ethics Committee of Huazhong University of Science and Technology. The methods of this study were carried out in accordance with the approved guidelines expressed in the Declaration of Helsinki.

Screening was done at the community health centres of the two districts. The sequence of events from recruitment to study exit is shown in Figure 1. On screening day, sociodemographic information and cervical cancer-related risk factors including reproductive and behavioural data were collected by trained health workers in confidential settings after informed written consent was obtained. Then cervical specimens for HPV test were collected from each participant by gynaecologists. One month later, all HPV positive women and 40% HPV negative women were telephonically informed to come back to undergo liquid-based cytology. Eligible women were randomly referred for colposcopy and a biopsy was performed if indicated. Health workers in each community health centre filled the clinical part of the questionnaire with the results of biopsy.

### Reverse dot blot HPV test

The cervical brushes were taken and immediately placed into a tube containing specimen transport medium (1mL). The samples were stored at −20°C until required for testing. The reverse dot blot HPV test can detect 13 high-risk HPV types: 16, 18, 31, 33, 35, 39, 45, 51, 52, 56, 58, 59, 68, and other 10 HPV types: 6, 11, 42, 43, 53, 66, 73, 81, 82 and 83 in cervical specimens. All detection procedures were performed according to the manufacturer's instructions for the reverse dot blot HPV test by the laboratory of Yaneng BIOscience (Shenzhen Co., Ltd. China). The performance of the reverse dot blot HPV test was approved by the China Food and Drug Administration.

### Liquid based cytology

Liquid-based cytology samples were collected by cervical cytobrush sampling, and placed into tube with 2 ml PreservCyt solution (U.S. Cyt Company) along with the cytobrush. The samples were stored at 4°C, and then sent to the laboratory for tests. ThinPrep slides were doubled-screened by cytotechnologists. Cytological results were reported using the 2001 Bethesda classification system and were classifies as [61, 62]: 1) negative; 2) atypical squamous cells of undetermined significance(ASCUS); 3) low-grade squamous intraepithelial lesion(LSIL); 4) atypical squamous cells that cannot exclude HSIL (ASC-H); 5) high-grade squamous intraepithelial lesion(HSIL).

### Colposcopy and histology

Colposcopy-guided tissue biopsies were taken from suspicious lesions by gynaecologists. Histology was examined and classified according to international criteria as follows [63]: normal, Cervical intraepithelial neoplasia (CIN) grade 1 (CIN 1), grade 2 (CIN 2), grade 3 (CIN 3), and invasive Squamous cell carcinoma (SCC).

### Statistical analysis

Clinical sensitivity, clinical specificity, positive predictive value (PPV), and negative predictive value (NPV) were computed using conventional contingency tables. The PPV means the risk of CIN2+ with a positive test. 1-NPV signifies the risk of CIN2+ with a negative test [64]. Thus, a low PPV would indicate the unnecessary procedures induced by cervical screening, and a low NPV reflects that the detection was not sufficient to exclude the potential disease. The 95% confidence intervals (95% CI) were calculated for proportions of sensitivity, specificity, PPV and NPV using the exact binomial method. Comparison of the HPV genotype representation between the different groups regarding categorical variables was done using the Chi-square test. Linear-by-linear association test was used to analyse trend in distribution of HPV infection according to grade of pathology abnormalities. All statistical tests were two-sided, *P* values <0.05 were considered statistically significant.

The gold standard in our study was histologically confirmed CIN2 or more severe diagnosis (CIN2, CIN3 and cancer). Cytology in our study was dichotomized, and a positive result was regarded as ASCUS or worse (ASCUS, LSIL, ASC-H, HSIL). To evaluate the triage strategies, we considered the NPV for CIN2+ of at least 98.0% to be acceptable [65] (corresponding with a <2% risk of CIN2+ within the next 2-3 years). Analyses were done using the SAS (version 9.1.3) and SPSS (version 12.0) software.

## Abbreviations

HPV: Human papillomavirus
PPV: Positive predictive value
NPV: Negative predictive value
CIN: Cervical intraepithelial neoplasia
SCC: Squamous cell carcinoma
NILM: No intraepithelial lesion or malignant cells
ASCUS: Atypical squamous cells of undetermined significance
LSIL: Low-grade squamous intraepithelial lesion
ASC-H: Atypical squamous cells that cannot exclude HSIL
HSIL: High-grade squamous intraepithelial lesion
CI: Confidence interval
SD: Standard deviation
